# Screening-Based Optimization of a Herbal Mixture (JH01) with Robust Anti-Obesity Effects in High-Fat Diet-Induced Obesity

**DOI:** 10.3390/ijms27073214

**Published:** 2026-04-01

**Authors:** Sung Jin Kim, Yea Jung Choi, Jong Hyun Hwang, Bokyung Lee, Gwi Seo Hwang

**Affiliations:** 1 College of Korean Medicine, Gachon University, Seongnam 13120, Republic of Korea; sungjinkim001@gmail.com (S.J.K.); domdada22@gachon.ac.kr (Y.J.C.); missionary88@naver.com (J.H.H.); 2 DAU G-LAMP Project Group, Innovation Center for Atomic Science Dong-A University, Busan 49315, Republic of Korea; 3 Department of Food Science and Nutrition, Dong-A University, Busan 49315, Republic of Korea; 4 Department of Health Sciences, The Graduate School of Dong-A University, Busan 49315, Republic of Korea

**Keywords:** obesity, herbal mixture, adipogenesis, lipid metabolism, high-fat diet

## Abstract

Obesity is a complex metabolic disorder associated with dyslipidemia, insulin resistance, and hepatic steatosis. Given its multifactorial nature, multi-component therapeutic strategies have attracted increasing interest, particularly herbal formulations containing diverse bioactive compounds. This study investigated the anti-obesity and hepatoprotective effects of a mixed herbal extract, JH01, composed of *Curcuma longa*, *Achyranthes bidentata*, and *Polygonum multiflorum*, using a screening-based analytical approach combined with experimental validation. Individual herbal extracts and their mixture were screened at 100 and 500 μg/mL in 3T3-L1 adipocytes. Based on superior anti-adipogenic efficacy, JH01 was selected for further study. Its effects were evaluated in vitro by Oil Red O staining and quantitative real-time PCR analysis of adipogenic genes, and in vivo using a high-fat diet (HFD)-induced obese mouse model, assessing body weight, serum lipid profiles, liver function markers, adipokine levels, and hepatic histology. JH01 showed markedly stronger inhibition of lipid accumulation than individual herbal components. JH01 significantly suppressed adipocyte differentiation and downregulated PPARγ, C/EBPα, and SREBP-1 expression in 3T3-L1 cells. Furthermore, JH01 modulated inflammatory cytokines and adipokine levels, as evidenced by reduced TNF-α, IL-6, and IL-1β levels and increased adiponectin levels. In HFD-fed mice, JH01 reduced body weight gain, serum triglyceride and total cholesterol levels, improved ALT and AST levels, decreased leptin concentrations, and attenuated hepatic steatosis. JH01 exerts potent anti-obesity and hepatoprotective effects through coordinated regulation of lipid metabolism and adipogenesis, supporting its potential as a multi-herbal therapeutic strategy for obesity-related metabolic disorders.

## 1. Introduction

Obesity has become a global health concern, with its prevalence increasing rapidly over recent decades [1,2]. It is a complex metabolic disorder characterized by excessive fat accumulation and is strongly associated with various comorbidities, including type 2 diabetes, cardiovascular disease, and non-alcoholic fatty liver disease (NAFLD) [3,4]. The pathogenesis of obesity involves dysregulation of lipid metabolism, chronic low-grade inflammation, insulin resistance, and abnormal adipokine secretion, highlighting the multifactorial nature of this condition [5].

Current pharmacological interventions for obesity primarily focus on appetite suppression, inhibition of nutrient absorption, or enhancement of energy expenditure [6,7]. However, these approaches often exhibit limited efficacy and are frequently associated with adverse effects, emphasizing the need for safer and more effective therapeutic strategies [8]. In this context, herbal medicines have attracted considerable attention due to their long history of use, structural diversity of bioactive compounds, and multi-target pharmacological properties [9].

*Curcuma longa*, *Achyranthes bidentata*, and *Polygonum multiflorum* are widely used in traditional East Asian medicine for the treatment of metabolic disorders, inflammatory diseases, and hepatic dysfunction [10,11,12]. Previous studies have reported that *C*. *longa* possesses anti-inflammatory, antioxidant, and lipid-lowering properties, while *A*. *bidentata* exhibits regulatory effects on lipid metabolism and bone health [13,14,15]. *P*. *multiflorum* has been traditionally used for liver protection and blood circulation improvement [16,17,18]. Despite these reported benefits, the molecular mechanisms underlying their potential synergistic effects remain poorly understood.

A screening- and target-oriented analytical approach has been increasingly applied to explore the multi-target mechanisms of herbal medicines [19]. By integrating compound screening, target prediction, and pathway-related analysis, this strategy enables the identification of key molecular targets and biological processes potentially modulated by multi-component herbal formulations [20]. In the present study, we employed a screening-based analytical strategy to investigate the anti-obesity potential of a mixed herbal formulation, designated as JH01, composed of *C*. *longa*, *A. bidentata*, and *P*. *multiflorum*. Bioactive compounds of *C*. *longa* and *A*. *bidentata* were screened using the TCMSP database, followed by target prediction and protein–protein interaction analysis. *P*. *multiflorum* was not included in the TCMSP database and was therefore excluded from the database analysis, which is acknowledged as a limitation of this study [21].

Subsequently, the anti-adipogenic effects of JH01 were evaluated in 3T3-L1 adipocytes, and its anti-obesity and hepatoprotective effects were further validated in a high-fat diet-induced obese mouse model. By integrating computational prediction with experimental validation, this study aims to provide a comprehensive understanding of the therapeutic potential and underlying mechanisms of JH01 in the management of obesity and obesity-related hepatic dysfunction.

This study employed an integrated research strategy combining network pharmacology and experimental validation to investigate the anti-obesity effects and underlying mechanisms of JH01 [22,23].

## 2. Results

### 2.1. Identification of Active Compounds in Curcuma longa and Achyranthes bidentata Using the TCMSP Database

To identify the bioactive compounds of Curcuma longa and Achyranthes bidentata, the Traditional Chinese Medicine Systems Pharmacology (TCMSP) database was utilized. Compounds were screened based on oral bioavailability (OB) and drug-likeness (DL) criteria. Specifically, compounds with OB ≥ 20% and DL ≥ 0.10 were selected as active compounds for further analysis.

As a result, multiple active compounds meeting these criteria were identified from both C. longa and A. bidentata. The detailed information, including compound names, OB values, and DL values, is summarized in Table 1 and Table 2, respectively. These selected compounds were subsequently used for target protein prediction and obesity-related relevance analysis.

### 2.2. Network Construction and Synergistic Target Analysis

A compound–target interaction network was constructed to analyze the relationship between the active compounds of Curcuma longa and Achyranthes bidentata and obesity-related molecular targets. Active compounds screened based on oral bioavailability and drug-likeness criteria were subjected to target prediction using the STITCH database. As a result, a total of ten target proteins were identified as common interaction nodes within the network.

**Figure 1:**
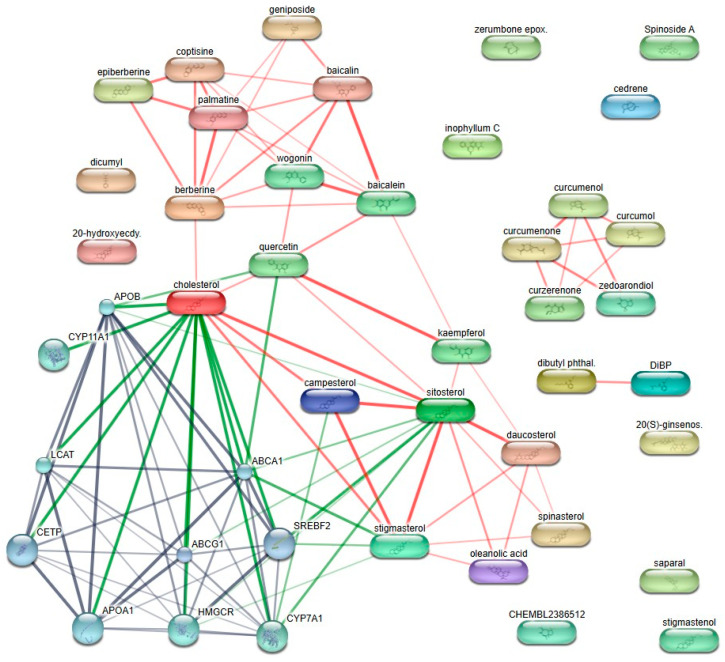
Predicted target proteins of *Curcumaelongae Rhizoma* and *Achyranthis Bidentatae Radix* active compounds. Edge colors indicate the type of evidence supporting the interaction: green for curated databases, red for text mining, and gray for experimental evidence.

The identified targets were APOA1, APOB, ABCA1, ABCG1, CETP, LCAT, HMGCR, CYP7A1, SREBF2, and CYP11A1. These proteins formed a densely connected target cluster, indicating strong functional associations among the predicted targets. Network visualization revealed that multiple compounds from both herbal sources converged on shared targets, suggesting overlapping target profiles between the two herbs (Figure 1).

### 2.3. Obesity Relevance Analysis of Target Proteins Using the GeneCards Database

**Figure 2:**
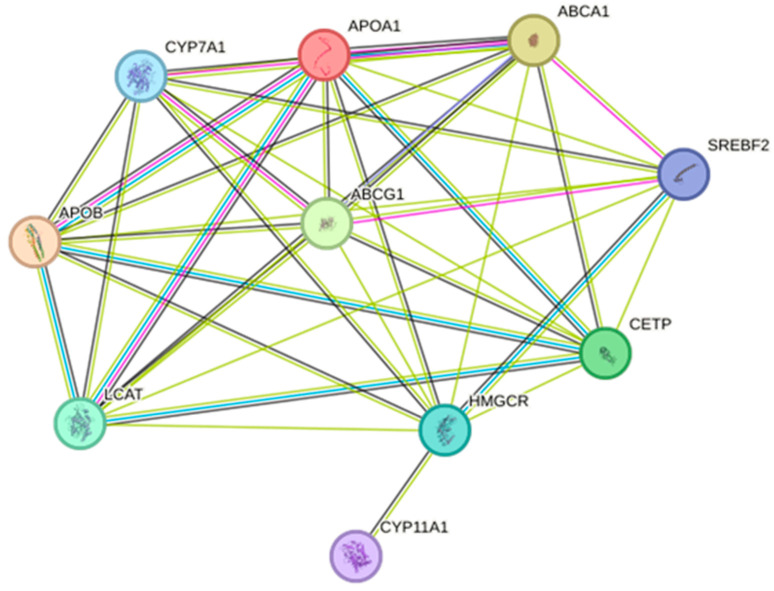
Common targets between *Curcumaelongae Rhizoma* and *Achyranthis Bidentatae* Radix compounds and obesity-related genes.

To evaluate the relevance of these targets to obesity, GeneCards database analysis was performed. All ten targets exhibited high relevance scores and GIFtS values, indicating well-characterized associations with obesity and lipid metabolism-related phenotypes (Figure 2). In particular, APOB, ABCA1, LCAT, and APOA1 showed high relevance scores, while CETP, HMGCR, SREBF2, and CYP7A1 also demonstrated strong obesity-related associations (Table 3).

### 2.4. Prediction of the Mechanisms of Action of Target Proteins from Curcumae Longae Rhizoma and Achyranthis Bidentatae Radix Using ClueGO

**Figure 3:**
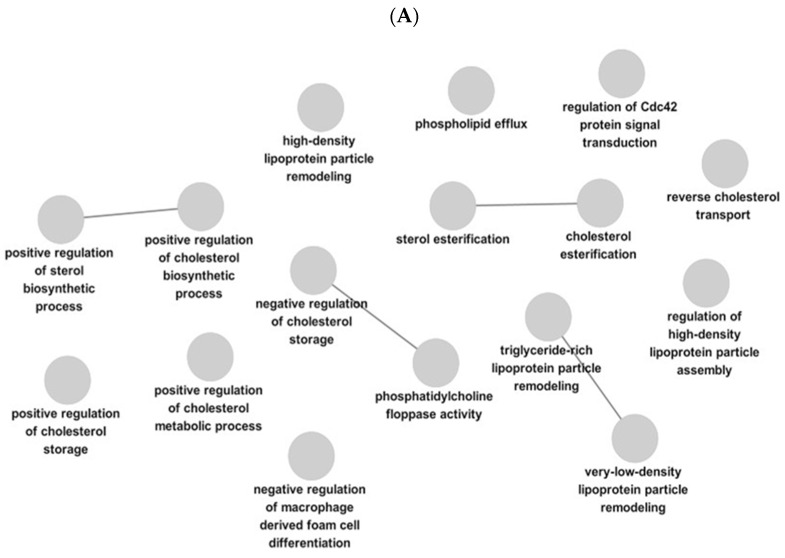

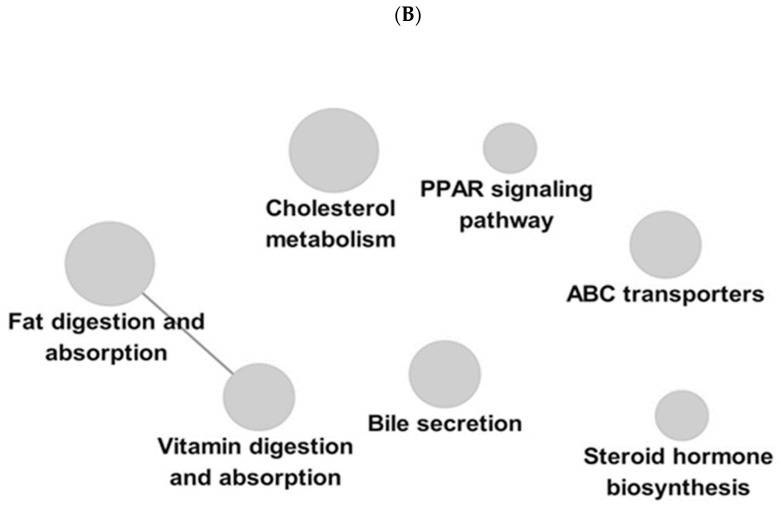
GO and KEGG pathway enrichment analysis of target proteins of *Curcumae Longae Rhizoma* and *Achyranthis Bidentatae Radix*. (**A**) GO biological process network analysis. Nodes represent significantly enriched biological processes, and edges indicate shared proteins between processes. The network was constructed using a manually defined significance threshold. (**B**) KEGG pathway enrichment analysis of the same target proteins. The top enriched pathways are presented based on significance, where node size reflects the number of involved target proteins and color intensity corresponds to the enrichment significance. Pathways related to inflammation, apoptosis, and metabolic regulation were predominantly enriched.

Gene Ontology (GO) biological process analysis revealed that the proteins identified in this study were significantly associated with biological processes related to cholesterol metabolism and lipid homeostasis. In particular, terms such as positive regulation of cholesterol biosynthetic process, positive regulation of cholesterol metabolic process, and positive regulation of cholesterol storage were enriched, suggesting that this gene set may be involved in regulatory mechanisms promoting cholesterol biosynthesis and storage (Figure 3A).

Additionally, processes related to cholesterol esterification and sterol esterification were significantly enriched, indicating a potential role in maintaining intracellular homeostasis through the esterification of free cholesterol. Regarding lipid transport, terms including reverse cholesterol transport, phospholipid efflux, as well as high-density lipoprotein (HDL) particle remodeling and very-low-density lipoprotein (VLDL) particle remodeling were identified, suggesting that this gene set may contribute to lipoprotein remodeling and the regulation of cholesterol transport (Figure 3B).

Consistent with these findings, KEGG pathway analysis also indicated the involvement of these genes in lipid metabolism-related pathways. Specifically, pathways such as cholesterol metabolism, the PPAR signaling pathway, ABC transporters, and bile secretion were enriched, which are associated with cholesterol transport, lipid absorption, and metabolic regulation.

Notably, the KEGG results showed a pattern consistent with the GO biological process analysis, with overlapping functional themes related to cholesterol metabolism and lipid transport. These findings suggest that the identified gene set may be functionally associated with the regulation of lipid homeostasis and cholesterol metabolism.

### 2.5. Preparation of JH01 Extract and Extraction Yield

A mixed herbal formulation, designated as JH01, composed of *Curcuma longa*, *Achyranthes bidentata*, and *Polygonum multiflorum*, was prepared. The dried raw materials were mixed at a ratio of 1:1:1 (*w*/*w*/*w*) and subjected to fermentation prior to extraction. The mixture was then extracted using 30% ethanol, followed by concentration under reduced pressure and freeze-drying. This extraction procedure was designed to ensure the recovery of both polar and moderately non-polar constituents from the herbal materials.

The extraction yields of individual herbal extracts and the mixed formulation were quantitatively evaluated (Table 4). The JH01 mixture exhibited a higher extraction yield than the corresponding single-herb extracts, indicating improved recovery of extractable components through the combined formulation.

To support the identification of bioactive compounds in the herbal formulation, TCMSP-based analysis was applied. Although TCMSP does not fully account for extraction conditions, it is widely used in herbal pharmacology studies to predict pharmacologically relevant compounds and their associated targets. The analysis revealed that multiple compounds derived from the individual herbal components were associated with obesity-related targets, reflecting the multi-component characteristics of the formulation [18,19,24].

### 2.6. JH01 Inhibits Adipocyte Differentiation in 3T3-L1 Cells

**Figure 4:**
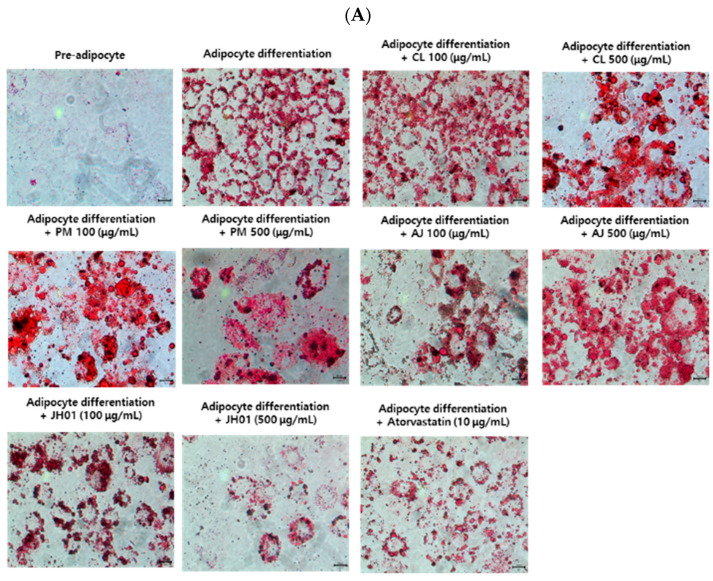

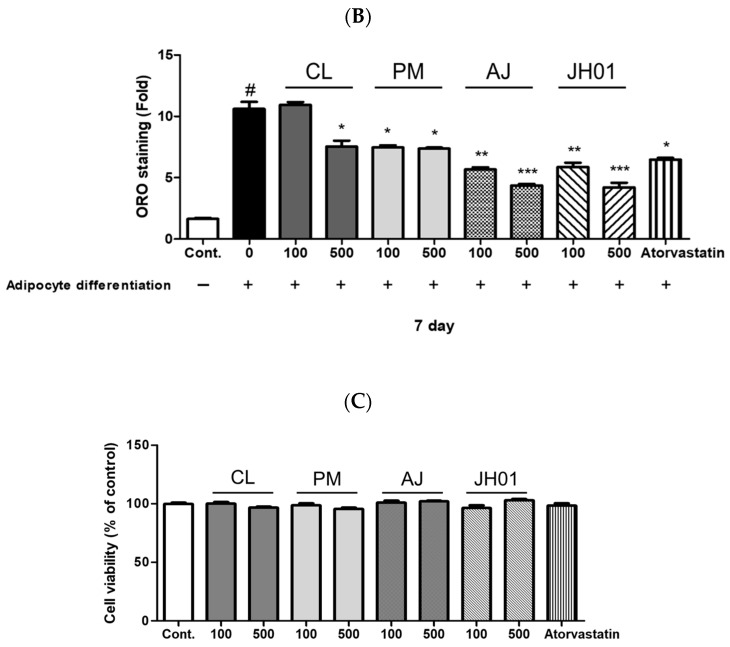
Inhibitory effects of JH01 and individual herbal extracts on adipocyte differentiation in 3T3-L1 cells. (**A**) 3T3-L1 preadipocytes were induced to differentiate and treated with the herbal mixture JH01 or individual herbal extracts, including *Curcuma longa* (CL), *Polygonum multiflorum* (PM), and *Achyranthes japonica* (AJ), at concentrations of 100 and 500 μg/mL. Lipid accumulation was assessed by Oil Red O (ORO) staining to evaluate the inhibitory effects on adipocyte differentiation. (**B**) Intracellular lipid accumulation was quantified by eluting ORO-stained lipid droplets with isopropanol, followed by absorbance measurement at 450 nm. (**C**) Cell viability was evaluated using the EZ-Cytox cell viability assay kit to exclude potential cytotoxic effects of JH01 and the individual herbal extracts. A scale bar of 100 μm (100× magnification) is indicated in the lower right corner. # *p* < 0.01 vs. cont. * *p* < 0.05, ** *p* < 0.01, and *** *p* < 0.001 vs. differentiated control group.

Oil Red O staining demonstrated that treatment with individual herbal extracts (*Curcuma longa*, *Achyranthes bidentata*, and *Polygonum multiflorum*) at 100 and 500 μg/mL resulted in partial inhibition of lipid accumulation in differentiated 3T3-L1 adipocytes. However, the magnitude of lipid reduction induced by the single-herb extracts was limited. In contrast, the mixed herbal formulation JH01 exhibited a markedly stronger inhibitory effect on lipid accumulation at the same concentrations (Figure 4A).

Quantitative analysis revealed that lipid accumulation was significantly reduced in the 100 and 500 μg/mL JH01-treated groups compared with the differentiation control, indicating superior anti-adipogenic activity of the mixed formulation relative to the individual herbal extracts. To assess whether the observed inhibition of lipid accumulation was associated with cytotoxic effects, cell viability was evaluated using the EZ-Cytox assay (Figure 4B) (Table S1).

Neither the individual herbal extracts nor JH01 showed significant cytotoxicity at 100 or 500 μg/mL, indicating that the anti-adipogenic effects were not attributable to reduced cell viability (Figure 4C) (Table S2).

### 2.7. JH01 Suppresses Adipogenic Gene Expression In Vitro

**Figure 5:**
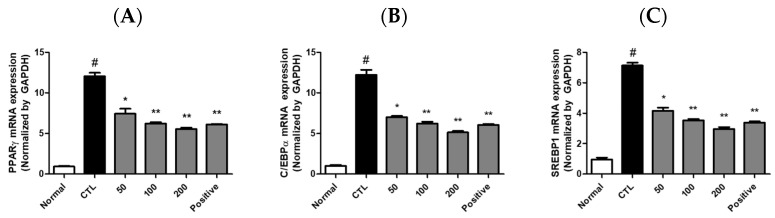
Effects of JH01 on adipogenesis-related gene expression in differentiated 3T3-L1 cells. Differentiated 3T3-L1 adipocytes were treated with JH01 during the adipogenic induction period. The mRNA expression levels of key adipogenic transcription factors were analyzed by quantitative real-time PCR (qPCR): (**A**) peroxisome proliferator-activated receptor gamma (Pparγ), (**B**) CCAAT/enhancer-binding protein alpha (Cebpa), and (**C**) sterol regulatory element-binding protein 1 (Srebp1). Gene expression levels were normalized to an internal control and are presented as relative expression levels compared with the differentiated control group. # *p* < 0.01 vs. cont. * *p* < 0.05, ** *p* < 0.01 vs. differentiated control group.

The mRNA expression levels of PPARγ, C/EBPα, and SREBP-1 were significantly downregulated by JH01 treatment. Relative expression levels of PPARγ were reduced to approximately 72 ± 6%, 48 ± 5%, and 31 ± 4% at 50, 100, and 200 μg/mL, respectively (Figure 5A). Similarly, C/EBPα expression was reduced to approximately 70 ± 7%, 46 ± 6%, and 34 ± 5%, while SREBP-1 expression was reduced to approximately 75 ± 6%, 52 ± 5%, and 38 ± 4% of the control level (Table S3).

### 2.8. JH01 Reduces Body Weight Gain in HFD-Induced Obese Mice

**Figure 6:**
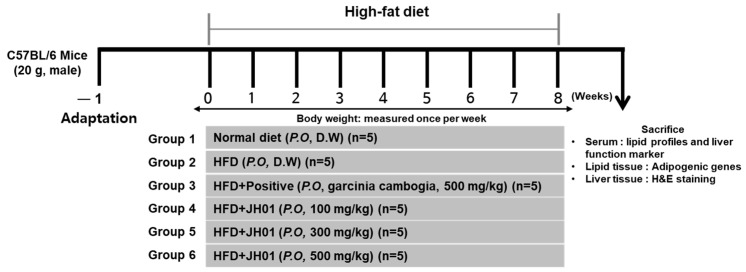
Schematic overview of the animal experimental protocol. Following a one-week acclimation period, mice were fed a normal diet or a high-fat diet to induce obesity. JH01 was orally administered to HFD-fed mice throughout the treatment period. Body weight was recorded weekly. At the end of the study, animals were fasted and sacrificed, and serum and tissue samples were harvested for downstream analyses.

After 8 weeks of high-fat diet (HFD) feeding, mice were administered JH01 at doses of 100, 300, and 500 mg/kg during the experimental period (Figure 6). The HFD group showed significantly greater body weight gain (approximately 7.6 ± 0.8 g) compared with the normal group (3.4 ± 0.6 g). In contrast, JH01 administration significantly attenuated body weight gain to approximately 5.8 ± 0.7 g, 4.6 ± 0.6 g, and 3.7 ± 0.5 g in the 100, 300, and 500 mg/kg groups, respectively (Table 5).

### 2.9. JH01 Modulates Leptin Levels and Adipogenic Markers in Adipose Tissue

**Figure 7:**
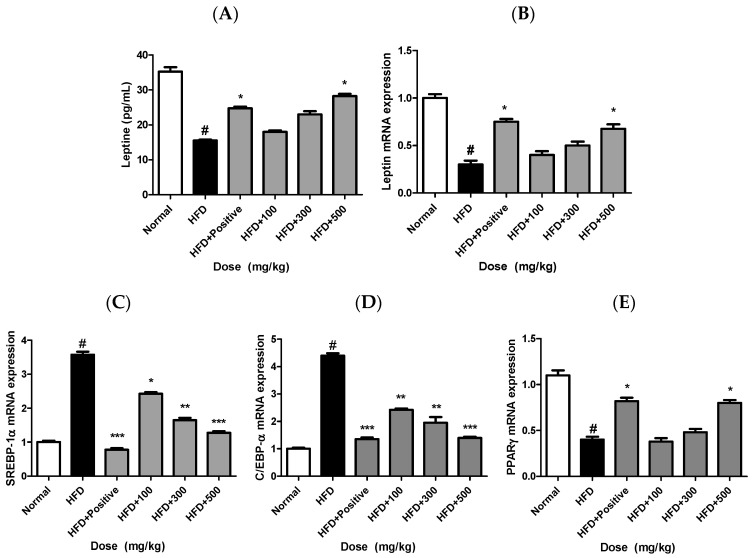
Effects of JH01 on adipogenesis- and adipokine-related gene expression. The mRNA and protein expression levels of adipogenic transcription factors and adipokines were analyzed following JH01 treatment: (**A**) leptin protein, (**B**) leptin mRNA, (**C**) sterol regulatory element-binding protein 1 (Srebp1), (**D**) CCAAT/enhancer-binding protein alpha (Cebpa), and (**E**) peroxisome proliferator-activated receptor gamma (Pparγ). Gene expression levels were normalized to an internal control and are presented as relative expression levels compared with the control group. # *p* < 0.01 vs. normal group. * *p* < 0.05, ** *p* < 0.01, and *** *p* < 0.001 vs. HFD group.

Serum leptin levels were markedly elevated in the HFD group compared with the normal group, indicating obesity-associated leptin dysregulation. JH01 treatment significantly reduced serum leptin levels in a dose-dependent manner (Figure 7A).

Consistent with the serum findings, leptin mRNA expression in adipose tissue was also significantly increased in the HFD group and was markedly suppressed by JH01 administration (Figure 7B).

In addition, the expression levels of key adipogenic transcription factors, including PPARγ, C/EBPα, and SREBP-1, were significantly upregulated in the adipose tissue of HFD-fed mice compared with the normal group. Treatment with JH01 resulted in a dose-dependent downregulation of these adipogenic markers (Figure 7C–E).

Overall, these results indicate that JH01 attenuates obesity-associated alterations in leptin production and adipogenic gene expression, suggesting its regulatory effects on adipose tissue function and lipid metabolism (Table S4).

### 2.10. JH01 Modulates Inflammatory Cytokines and Adipokine Levels in Adipose Tissue

**Figure 8:**
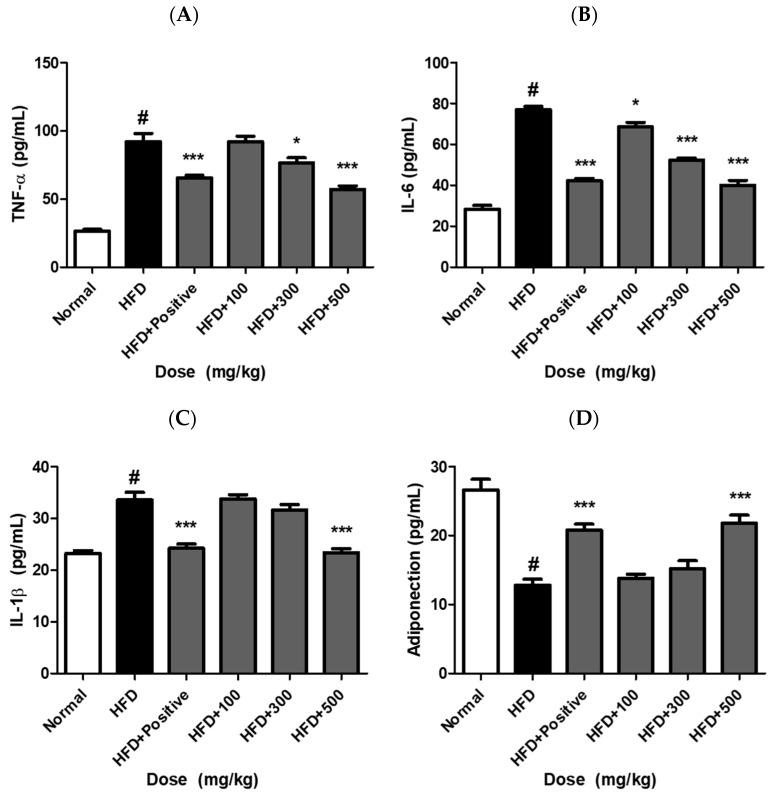
JH01 modulates inflammatory cytokines and adipokine levels in adipose tissue. Levels of pro-inflammatory cytokines (TNF-α, IL-6, and IL-1β) and the anti-inflammatory adipokine adiponectin were measured in adipose tissue of mice fed a normal diet, HFD, or HFD supplemented with JH01 at different doses (**A**–**C**). The HFD group exhibited increased levels of TNF-α, IL-6, and IL-1β, along with decreased adiponectin levels (**D**), indicating adipose tissue inflammation. JH01 treatment attenuated the levels of pro-inflammatory cytokines and restored adiponectin expression in a dose-dependent manner. Data are presented as mean ± SEM. # *p* < 0.0001 vs. normal group. * *p* < 0.05, *** *p* < 0.0001 vs. HFD group.

In adipose tissue, pro-inflammatory cytokines such as TNF-α, IL-6, and IL-1β function as key adipokines that mediate obesity-induced inflammation, whereas adiponectin acts as an anti-inflammatory adipokine that contributes to metabolic homeostasis [4,25]. To evaluate the anti-inflammatory and metabolic regulatory effects of JH01, the levels of these cytokines and adipokines were assessed. The HFD group exhibited significantly elevated levels of TNF-α, IL-6, and IL-1β compared with the normal group, indicating the induction of adipose tissue inflammation under obese conditions (Figure 8A–C). In contrast, adiponectin levels were markedly reduced in the HFD group (Figure 8D). JH01 treatment significantly attenuated these inflammatory responses in a dose-dependent manner. The levels of TNF-α and IL-6 were substantially decreased in the JH01-treated groups, whereas IL-1β showed a moderate reduction. Conversely, adiponectin levels were significantly increased following JH01 treatment, showing a dose-dependent recovery toward normal levels.

These findings suggest that JH01 effectively suppresses obesity-induced inflammatory responses in adipose tissue while restoring adipokine balance, thereby contributing to improved metabolic homeostasis (Table S5).

### 2.11. JH01 Improves Serum Lipid Profiles and Liver Function

**Figure 9:**
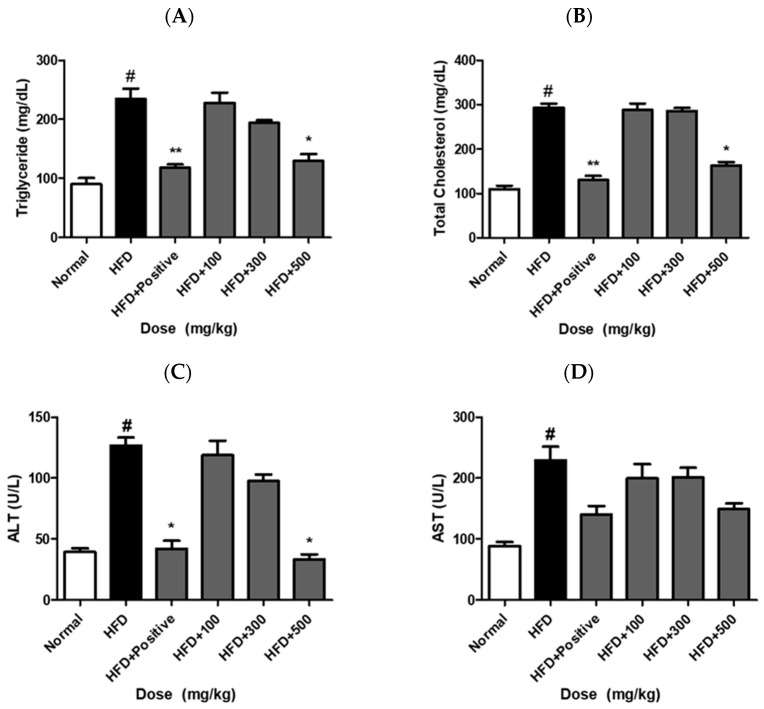
Effects of JH01 on serum lipid profiles and liver function markers. Serum levels of triglycerides (TG) and total cholesterol (TC) were measured to evaluate lipid metabolism (**A**,**B**). In addition, serum alanine aminotransferase (ALT) and aspartate aminotransferase (AST) levels were assessed as indicators of liver function (**C**,**D**). # *p* < 0.01 vs. normal group. * *p* < 0.05, and ** *p* < 0.01, vs. HFD group.

Serum TG levels were increased to approximately 182 ± 15 mg/dL in the HFD group compared with 96 ± 12 mg/dL in the normal group. JH01 treatment significantly reduced TG levels to approximately 152 ± 13 mg/dL, 131 ± 11 mg/dL, and 114 ± 10 mg/dL in the low-, middle-, and high-dose groups, respectively (Figure 9A). Similarly, TC levels were reduced from approximately 215 ± 18 mg/dL (HFD) to 182 ± 16 mg/dL, 164 ± 14 mg/dL, and 142 ± 13 mg/dL following JH01 treatment (Figure 9B) (Table S6).

### 2.12. JH01 Reduces Hepatic Lipid Deposition in HFD-Induced Obese Mice

**Figure 10:**
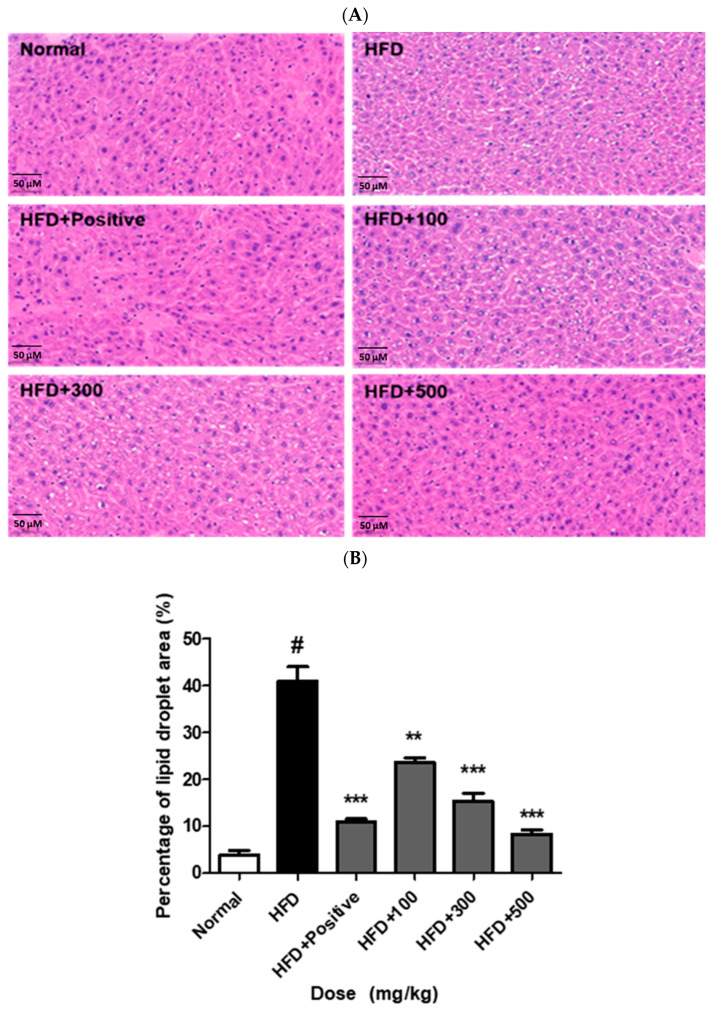
JH01 attenuates HFD-induced hepatic lipid accumulation. (**A**) Representative hematoxylin and eosin (H&E)-stained sections of liver tissues from mice fed a normal diet (ND), a high-fat diet (HFD), or a high-fat diet supplemented with JH01. The HFD group exhibited marked hepatic steatosis characterized by extensive lipid droplet accumulation, whereas JH01-treated mice showed a noticeable reduction in lipid droplets and improved hepatic architecture. (**B**) Quantitative analysis of hepatic lipid accumulation expressed as the percentage of lipid droplet area relative to the total tissue area. Lipid droplet area was quantified using ImageJ software (version 1.53, National Institutes of Health, Bethesda, MD, USA). # *p* < 0.01 vs. normal group. ** *p* < 0.01, and *** *p* < 0.001 vs. HFD group. Scale bar = 50 μm.

Histological analysis of liver tissues was performed using hematoxylin and eosin (H&E) staining to evaluate hepatic lipid accumulation. As shown in Figure 10A, mice in the HFD group exhibited increased lipid droplet accumulation in hepatocytes compared with the ND group. In contrast, the JH01-treated groups showed reduced lipid droplet accumulation compared with the HFD group. Quantitative analysis confirmed that the lipid droplet area was significantly increased in the HFD group compared with the ND group (Figure 10B). Treatment with JH01 significantly decreased the hepatic lipid droplet area compared with the HFD group. These findings indicate that JH01 administration was associated with reduced hepatic lipid accumulation in HFD-fed mice (Table S7).

## 3. Discussion

In the present study, we demonstrated that the screening-based herbal formulation JH01, composed of *Curcuma longa*, *Achyranthes bidentata*, and *Polygonum multiflorum*, exerts robust anti-obesity and hepatoprotective effects through coordinated regulation of adipogenesis, lipid metabolism, and hepatic lipid accumulation. These findings extend previous reports on the individual metabolic benefits of each herbal component by providing experimental evidence for their enhanced efficacy when combined into a single optimized formulation. Previous studies have reported that *Curcuma longa*, particularly its major bioactive compound curcumin, suppresses adipocyte differentiation and lipid accumulation primarily through inhibition of PPARγ and C/EBPα signaling pathways, as well as activation of AMPK-mediated lipid catabolism [26,27,28]. Similarly, *Achyranthes bidentata* has been shown to regulate lipid metabolism and improve metabolic homeostasis by modulating adipogenic gene expression and inflammatory responses in adipose tissue [29,30]. Polygonum multiflorum has been traditionally used for hepatic protection, and experimental studies have demonstrated its ability to attenuate hepatic steatosis and oxidative stress in diet-induced metabolic disorder models [31,32].

While these studies highlight the therapeutic potential of individual herbs, their effects are often moderate when administered alone. In contrast, our screening results revealed that JH01 inhibited lipid accumulation in 3T3-L1 adipocytes more effectively than any single extract at equivalent concentrations. This suggests that the observed biological activity of JH01 is not merely additive, but rather arises from complementary or synergistic interactions among its components. Such synergy is a fundamental characteristic of multi-herbal formulations and has been increasingly recognized as a key advantage over single-compound interventions in metabolic disease management [18,33].

Adipocyte differentiation is tightly regulated by a network of transcription factors, among which PPARγ, C/EBPα, and SREBP-1 play central roles. PPARγ is considered the master regulator of adipogenesis, governing adipocyte maturation, lipid uptake, and insulin sensitivity [34]. C/EBPα cooperates with PPARγ to establish and maintain the differentiated adipocyte phenotype, while also regulating genes involved in glucose and lipid metabolism [35]. SREBP-1 primarily controls de novo lipogenesis by regulating fatty acid synthase (FASN), acetyl-CoA carboxylase (ACC), and other lipogenic enzymes [36].

In this study, JH01 significantly downregulated the expression of PPARγ, C/EBPα, and SREBP-1 in a dose-dependent manner, indicating that JH01 interferes with adipogenic programming at multiple regulatory levels. Compared to previous studies focusing on single herbal extracts that typically target one dominant pathway, the simultaneous suppression of these key transcriptional regulators suggests a broader and more integrated mechanism of action [26,29,34]. This multi-target regulation likely contributes to the superior anti-adipogenic efficacy observed for JH01.

Consistent with the in vitro findings, JH01 administration significantly attenuated body weight gain and improved lipid profiles in HFD-induced obese mice. Reductions in serum triglyceride and total cholesterol levels indicate an overall improvement in systemic lipid metabolism. Similar lipid-lowering effects have been reported for *Curcuma longa* and *Achyranthes bidentata* individually. However, these effects often require relatively high doses or prolonged administration [27,30]. The pronounced metabolic improvements observed with JH01 at the tested dose further support the hypothesis of synergistic efficacy.

Leptin is a key adipokine involved in appetite regulation and energy homeostasis, and elevated circulating leptin levels are indicative of leptin resistance in obesity [37]. The significant reduction in serum leptin levels observed in JH01-treated mice suggests an improvement in adipose tissue function and leptin sensitivity. This finding is consistent with previous reports showing that suppression of adipogenesis and adipocyte hypertrophy leads to normalization of adipokine secretion patterns [38].

In adipose tissue, pro-inflammatory cytokines such as TNF-α, IL-6, and IL-1β act as key adipokines that contribute to obesity-associated inflammation, whereas adiponectin functions as an anti-inflammatory adipokine involved in maintaining metabolic homeostasis [25]. In the present study, JH01 treatment significantly reduced the levels of TNF-α, IL-6, and IL-1β while restoring adiponectin levels in adipose tissue. This coordinated regulation of pro- and anti-inflammatory adipokines suggests that JH01 improves adipokine balance, which is a critical factor in the amelioration of metabolic dysfunction associated with obesity [4].

Obesity-associated hepatic dysfunction is characterized by excessive lipid accumulation, inflammation, and elevated liver enzyme levels. In the present study, JH01 significantly reduced serum ALT and AST levels and markedly improved hepatic histological features, including reduced lipid droplet accumulation. These findings align with previous studies demonstrating the hepatoprotective effects of *Polygonum multiflorum* and Curcuma longa through attenuation of oxidative stress and lipid overload [31,32,39].

Importantly, the combined improvement in hepatic histology and serum biochemical markers suggests that JH01 not only prevents lipid accumulation but also alleviates metabolic stress in the liver. Given the close pathological relationship between obesity and non-alcoholic fatty liver disease (NAFLD), these hepatoprotective effects substantially enhance the clinical relevance of JH01 as a potential therapeutic strategy for obesity-related metabolic disorders [31].

Although target prediction and interaction analyses were employed to support the biological relevance of the screened components, this study prioritized experimental validation over predictive modeling. The exclusion of Polygonum multiflorum from database-driven analyses due to limited data availability represents a limitation. Nevertheless, its inclusion in the experimental formulation reflects real-world herbal practice and highlights the importance of empirical screening strategies in multi-herbal research [18,33].

Overall, our findings demonstrate that JH01 exerts significant anti-obesity and hepatoprotective effects through coordinated regulation of adipogenesis and lipid metabolism. By integrating experimental screening with mechanistic validation, this study provides a practical framework for the development of optimized, evidence-based multi-herbal formulations for obesity and obesity-related metabolic diseases.

## 4. Materials and Methods

### 4.1. JH01 Alleviates Hepatic Steatosis

Active compounds of Curcuma longa and Achyranthes bidentata were retrieved from the Traditional Chinese Medicine Systems Pharmacology (TCMSP) database (https://tcmsp-e.com/, accessed on 15 January 2026). Compounds were screened based on oral bioavailability (OB) and drug-likeness (DL) criteria. For both *C. longa* and *A. bidentata*, compounds with OB ≥ 20% or DL ≥ 0.10 were selected to ensure sufficient coverage of potentially bioactive constituents. Corresponding putative targets of the selected compounds were obtained from TCMSP and standardized using the UniProt database. Obesity-related genes were collected from GeneCards and OMIM databases. Overlapping targets between compound-related targets and obesity-related genes were identified and used for subsequent network analysis.

### 4.2. Screening-Based Target Association and Interaction Analysis

To investigate the molecular mechanisms of the selected herbal compounds, a multi-step network pharmacology approach was employed. First, active compounds and their associated proteins were retrieved from the STITCH database (https://string-db.org/, accessed on 19 January 2026). To refine these targets, the candidate proteins were further evaluated using the GeneCards database (https://www.genecards.org/, accessed on 21 January 2026), considering both the GeneCards Score and GIFtS metrics to select core proteins with high relevance.

Subsequently, protein–protein interaction (PPI) information for the selected core proteins was obtained from the STRING database (https://string-db.org/, accessed on 22 January 2026; confidence score ≥ 0.4) to examine potential functional associations among them. The resulting network was analyzed descriptively using network parameters such as degree and betweenness centrality, without performing hub-driven target selection.

Finally, the function and biological processes of the STRING-associated proteins were investigated using ClueGO in Cytoscape (ver.3.10.4), enabling visualization of enriched Gene Ontology (GO) biological processes.

### 4.3. Preparation of Herbal Extracts and Formulation of JH01

Dried raw materials of *Curcuma longa* L. (rhizome), *Achyranthes bidentata* Blume (root), and *Polygonum multiflorum* Thunb. (root) were purchased from a commercial herbal supplier in Kyungdong herbal market (Seoul, Republic of Korea) and authenticated by an expert in pharmacognosy. The plant materials were pulverized into a fine powder and mixed at a ratio of 1:1:1 (*w*/*w*/*w*). For the preparation of the herbal formulation JH01, the powdered mixture was subjected to yeast fermentation prior to extraction. Briefly, the herbal mixture was suspended in distilled water and sterilized before inoculation. A yeast strain (Saccharomyces cerevisiae) was inoculated at a final concentration of approximately 1 × 10^7^ CFU/mL. Fermentation was carried out at 30 °C for 48 h under aerobic conditions with continuous agitation. During fermentation, the pH and temperature were monitored and maintained within a controlled range. After completion of fermentation, the fermented mixture (JH01) was extracted with 30% (*v*/*v*) ethanol at 60 °C for 3 h under continuous stirring. The extract was filtered and concentrated under reduced pressure using a rotary evaporator, followed by freeze-drying to obtain the fermented JH01 extract powder. The extraction yield (%) was calculated as follows:

Yield (%) = (Weight of dried extract/Weight of dried raw material) × 100

### 4.4. Cell Culture and Adipocyte Differentiation

3T3-L1 preadipocytes were obtained from the American Type Culture Collection (ATCC, Manassas, VA, USA) and maintained in Dulbecco’s Modified Eagle’s Medium (DMEM; Thermo Fisher Scientific, Waltham, MA, USA) supplemented with 10% bovine calf serum (Thermo Fisher Scientific, Waltham, MA, USA) and 1% penicillin–streptomycin (Thermo Fisher Scientific, Waltham, MA, USA) at 37 °C in a humidified atmosphere containing 5% CO_2_.

For adipocyte differentiation, confluent cells were treated with differentiation medium containing 0.5 mM 3-isobutyl-1-methylxanthine (IBMX; Sigma-Aldrich, St. Louis, MO, USA), 1 μM dexamethasone (Sigma-Aldrich, St. Louis, MO, USA), and 10 μg/mL insulin (Sigma-Aldrich, St. Louis, MO, USA) for 2 days, followed by insulin-containing medium for an additional 2 days. Cells were then maintained in DMEM supplemented with 10% fetal bovine serum (FBS; Thermo Fisher Scientific, Waltham, MA, USA) [40,41].

### 4.5. Oil Red O Staining

Lipid accumulation in differentiated 3T3-L1 cells was assessed by Oil Red O staining. Cells were fixed with 10% formalin solution (Sigma-Aldrich, St. Louis, MO, USA) for 1 h, washed with distilled water, and stained with Oil Red O solution (Sigma-Aldrich, St. Louis, MO, USA) for 30 min. After washing, images were captured using a light microscope.

For quantitative analysis, the stained lipid droplets were eluted with isopropanol (Sigma-Aldrich, St. Louis, MO, USA), and absorbance was measured at 520 nm using a microplate reader.

### 4.6. Quantitative Real-Time PCR (qRT-PCR)

Total RNA was isolated using the RNeasy Mini Kit (Qiagen, Hilden, Germany) according to the manufacturer’s instructions. The concentration and purity of RNA were assessed spectrophotometrically. Complementary DNA (cDNA) was synthesized using a reverse transcription kit (Thermo Fisher Scientific, Waltham, MA, USA) following the manufacturer’s protocol [42].

Quantitative real-time PCR was performed using SYBR Green Master Mix (Thermo Fisher Scientific, Waltham, MA, USA) on a real-time PCR system. The relative mRNA expression levels of PPARγ, C/EBPα, and SREBP-1 were normalized to GAPDH as an internal control and calculated using the 2^−ΔΔCt^ method [22]. Primer sequences used for qRT-PCR are listed in Table 6.

### 4.7. Animal Experiments

All animal experimental procedures were approved by the Institutional Animal Care and Use Committee (IACUC) of Gachon University and were conducted in accordance with the guidelines for the care and use of laboratory animals. The IACUC approval number for this study was GU1-2022-IA0004-00.

Male C57BL/6J mice (6 weeks old) were obtained from OrientBio (Seongnam, Republic of Korea) and housed under standard laboratory conditions (22 ± 2 °C, 12 h light/dark cycle) with free access to food and water. After a one-week acclimatization period, the mice were randomly divided into the following groups: normal diet (ND), high-fat diet (HFD), HFD + JH01 (100 mg/kg), HFD + JH01 (300 mg/kg), HFD + JH01 (500 mg/kg), and HFD + Garcinia cambogia extract (500 mg/kg) as a positive control. The experimental diets were commercially obtained from DBL Co., Ltd. (Eumseong, Republic of Korea).

The experimental diets were purchased from DBL Co., Ltd. (Eumseong, Republic of Korea), and their detailed compositions are presented in Table 7. The HFD provided 60% of total energy from fat, whereas the ND contained 10% fat-derived energy.

JH01 and Garcinia cambogia extract were administered orally once daily for 8 weeks. Body weight and food intake were recorded weekly throughout the experimental period [43].

### 4.8. Serum Biochemical Analysis

Blood samples were collected from mice at the time of sacrifice and allowed to clot at room temperature. Serum was obtained by centrifugation at 3000× *g* for 15 min at 4 °C. Serum biochemical parameters, including triglycerides, total cholesterol, alanine aminotransferase, and aspartate aminotransferase, were analyzed using commercially available enzymatic assay kits. All serum biochemical analyses were performed by OvEn Co., Ltd. (Suwon, Gyeonggi-do, Republic of Korea) according to standardized protocols [44].

### 4.9. Histological Analysis of Liver Tissue

Liver tissues were excised immediately after sacrifice and fixed in 10% neutral-buffered formalin. Paraffin embedding, sectioning (4–5 μm thickness), and hematoxylin and eosin (H&E) staining were conducted by OvEn Co., Ltd. (Suwon, Gyeonggi-do, Republic of Korea) following standard histological procedures. Histological features, including hepatic lipid accumulation and tissue architecture, were examined and imaged using a light microscope [45]. Lipid accumulation was quantified using ImageJ software.

### 4.10. Statistical Analysis

All data are presented as the mean ± standard error of the mean (SEM). Statistical analyses were performed using GraphPad Prism (64-bit version; GraphPad Software, San Diego, CA, USA). Comparisons among multiple groups were conducted using one-way analysis of variance (ANOVA) followed by Tukey’s post hoc test. A *p*-value < 0.05 was considered statistically significant [46].

## 5. Conclusions

In conclusion, this study demonstrates that the optimized herbal mixture JH01, composed of Curcuma longa, Achyranthes bidentata, and Polygonum multiflorum, exerts significant anti-obesity and hepatoprotective effects through coordinated regulation of adipogenesis and lipid metabolism. JH01 was selected through experimental screening and showed superior efficacy compared with individual herbal components. JH01 effectively inhibited adipocyte differentiation by suppressing key adipogenic transcription factors, including PPARγ, C/EBPα, and SREBP-1, and attenuated obesity-associated metabolic alterations in an HFD-induced mouse model. These findings support the therapeutic potential of screening-based multi-herbal formulations and provide experimental evidence for the development of evidence-based herbal strategies for the management of obesity-related metabolic disorders.

## Figures and Tables

**Table 1:** Active compounds of *Curcumaelongae Rhizoma* screened by OB and DL criteria.

No.	Compound Name	OB (%)	DL
1	Stigmasterol	43.83	0.76
2	Campesterol	37.58	0.71
3	Cholesterol	37.87	0.68
4	Curcumenone	34.17	0.11
5	α-Cedrene	55.56	0.10
6	Curzerenone	57.05	0.11
7	Curcumenol	87.82	0.13
8	Curcumol	103.55	0.13
9	Zerumbone epoxide	27.26	0.11
10	4-Methoxy-5-hydroxybisabolene	53.67	0.11
11	3-Hydroxy-α-atlantone	46.11	0.10
12	Epiprocurcumenol	25.01	0.10
13	Zedoarondiol	59.37	0.12
14	Procurcumadiol	69.82	0.13
15	Bisacurone	36.92	0.10
16	Dicumene	38.08	0.11

**Table 2:** Active compounds of *Achyranthis Bidentatae Radix (Niuxi)* screened by OB and DL criteria.

No.	Compound Name	OB (%)	DL
1	Chondrillasterol	42.98	0.76
2	Ginsenoside Rg2	20.12	0.82
3	Achyranthoside D trimethyl ester	67.97	0.16
4	28-Norolean-17-en-3-one	35.93	0.78
5	Ecdysterone glucoside	21.20	0.63
6	Achyranthoside IV	20.86	0.11
7	Achyranthoside C	66.62	0.18
8	Achyranthoside C dimethyl ester	71.87	0.16
9	Achyranthoside E	24.15	0.20
10	Bidentatoside I	23.85	0.11
11	Bidentatoside II	31.76	0.59
12	Chikusetsusaponin-Iva Butyl Ester	52.25	0.11
13	Spinoside A	41.75	0.40
14	β-Ecdysterone	44.23	0.82
15	Berberine	36.86	0.78
16	Coptisine	30.67	0.86
17	Wogonin	30.68	0.23
18	Oleanolic acid	29.02	0.76
19	Delta-7-stigmastenol	37.42	0.75
20	Baicalein	33.52	0.21
21	Baicalin	40.12	0.75
22	Epiberberine	43.09	0.78
23	Sitogluside	20.63	0.62
24	β-Sitosterol	36.91	0.75
25	Geniposide	39.71	0.10
26	Inophyllum E	38.81	0.85
27	Kaempferol	41.88	0.24
28	Alpha-Spinasterol	42.98	0.76
29	Stigmasterol	43.83	0.76
30	Spinasterol 3-O-β-D-glucopyranoside	21.20	0.63
31	Diisobutyl phthalate	49.63	0.13
32	Dibutyl phthalate	64.54	0.13
33	Palmatine	64.60	0.65
34	β-Daucosterol	36.91	0.75
35	Quercetin	46.43	0.28

**Table 3:** Obesity-related target genes identified from GeneCards analysis.

Name	Obesity Score (GeneCards)	GIFtS
APOA1	91.1	62
APOB	135.2	58
ABCA1	126.8	61
ABCG1	83.9	55
CETP	98.2	60
LCAT	126.2	59
HMGCR	74.82	60
CYP7A1	67.1	54
SREBF2	57.86	55
CYP11A1	106.72	61

**Table 4:** Extraction yields of individual herbal extracts and the mixed formulation JH01.

Scientific Name	Solvent	Extraction Yield (%)
*Polygonum multiflorum*	30% EtOH	16.8
*Curcuma longa*	30% EtOH	19.5
*Achyranthes bidentata*	30% EtOH	15.9
Mixture (JHO1-N)	30% EtOH	23.4

**Table 5:** Effects of JH01 on body weight gain in high-fat diet-fed mice.

	Initial Weight (g)	Final Weight (g)	Body Weight Gain (g)
Normal	19.6 ± 0.5	22.8 ± 0.5	3.2
HFD	19.3 ± 0.3	26.8 ± 0.8	7.5 #
HFD + Positive	20.6 ± 0.2	24.3 ± 0.2	3.8 *
HFD + 100 mg/kg	20.5 ± 0.3	24.6 ± 0.3	4.1
HFD + 300 mg/kg	20.8 ± 0.5	25.2 ± 0.2	4.4
HFD + 500 mg/kg	20.4 ± 0.3	23.8 ± 0.4	3.5 *

# *p* < 0.01 vs. Normal group, and * *p* < 0.05 HFD group.

**Table 6:** Primer sequences used for qRT-PCR.

Gene	Accession No.	Primer Sequence (5 → 3)
PPARγ	NM_011146.3	F: GAAAGACAACGGACAAATCAC R: TACGGATCGAAACTGGCACC
C/EBPα	NM_007678.3	F: TGGACAAGAACAGCAACGAG R: TCACTGGTCAACTCCAGCAC
SREBP-1	NM_011480.3	F: GGAGCCATGGATTGCACATT R: GGCCCGGGAAGTCACTGT
GAPDH	NM_008084.3	F: AGGTCGGTGTGAACGGATTTG R: TGTAGACCATGTAGTTGAGGTCA

**Table 7:** Nutritional composition of ND and HFD.

Parameter	NFD	HFD
Protein (kcal%)	20	20
Carbohydrate (kcal%)	70	20
Fat (kcal%)	10	60
Energy density (kcal/g)	3.85	5.24
Cholesterol (mg/4057 kcal)	54.4	232.8

## Data Availability

The original contributions presented in this study are included in the article/Supplementary Materials. Further inquiries can be directed to the corresponding authors.

## Supplementary Materials

The following supporting information can be downloaded at: https://www.mdpi.com/article/10.3390/ijms27073214/s1.
