# Supplementary material for: Screening-Based Optimization of a Herbal Mixture (JH01) with Robust Anti-Obesity Effects in High-Fat Diet-Induced Obesity
Source: Int J Mol Sci. 2026 Apr 1;27(7):3214. doi: 10.3390/ijms27073214 (PMC13073794; doi:10.3390/ijms27073214)
Supplement: Supplementary file 1 [file ijms-27-03214-s001.zip › ijms-4182287-supplementary.pdf]

Supplementary Table S1. Quantification of Oil Red O (ORO) staining in differentiated adipocytes

Group	Dose (µg/mL)	ORO staining (fold of control)	SD
Control	0	1.0	0.2
Adipocyte differentiation	0	12.1	1.2
CL	100	12.3	0.6
CL	500	7.8	0.4
PM	100	7.6	0.3
PM	500	7.5	0.3
AJ	100	6.4	0.2
AJ	500	5.3	0.3
JH01	100	6.8	0.6
JH01	500	5.2	0.4
Atorvastatin	0	7.2	0.2

Values are presented as mean ± SD. Oil Red O (ORO) staining was quantified and expressed as fold change relative to the control group.

Supplementary Table S2. Effects of samples on cell viability

Group	Dose (μg/mL)	Cell viability (% of control)	SD
Control	0	100.3	0.2
CL	100	101.4	0.3
CL	500	98.7	0.2
PM	100	102.5	0.1
PM	500	99.5	0.4
AJ	100	101.4	0.2
AJ	500	102.6	0.3
JH01	100	98.6	0.2
JH01	500	103.2	0.5
Atorvastatin	0	101.6	0.2

Values are presented as mean ± SD. Cell viability was expressed as a percentage relative to the control group.

supplementary Table S3. Relative mRNA expression of PPAR-γ, C/EBP-α, and SREBP1 in 3T3-L1 adipocytes

Group	Dose (µg/mL)	Relative expression (fold of normal)	SD
Normal	–	0.933	0.153
Control	–	12.067	0.702
Treatment	50	7.433	1.050
Treatment	100	6.233	0.252
Treatment	200	5.533	0.306
Positive	–	6.100	0.100

Group	Dose (µg/mL)	Relative expression (fold of normal)	SD
Normal	–	1.000	0.200
Control	–	12.200	1.114
Treatment	50	7.033	0.208
Treatment	100	6.233	0.379
Treatment	200	5.133	0.306
Positive	–	6.067	0.153

Group	Dose (µg/mL)	Relative expression (fold of normal)	SD
Normal	–	0.967	0.208
Control	–	7.133	0.306
Treatment	50	4.167	0.351
Treatment	100	3.533	0.153
Treatment	200	2.967	0.208
Positive	–	3.367	0.153

Values are presented as mean ± SD. Gene expression levels were normalized to the normal group.

Supplementary Table S4. Serum leptin levels, Leptin, SREBP-1α, C/EBP-α, and PPAR-γ mRNA mRNA expression in adipose tissue

Group	Leptin (ng/mL)	SD
Normal	35.25	2.50
HFD	15.50	0.58
HFD + Positive	24.75	0.96
HFD + 100	18.00	0.82
HFD + 300	23.00	1.83
HFD + 500	28.25	1.26

Group	Relative expression (fold of normal)	SD
Normal	1.000	0.082
HFD	0.300	0.082
HFD + Positive	0.750	0.058
HFD + 100	0.400	0.082
HFD + 300	0.500	0.082
HFD + 500	0.675	0.096

Group	Relative expression (fold of normal)	SD
Normal	1.000	0.082
HFD	3.575	0.171
HFD + Positive	0.775	0.096
HFD + 100	2.425	0.096
HFD + 300	1.650	0.129
HFD + 500	1.275	0.096

Group	Relative expression (fold of normal)	SD
Normal	1.000	0.082
HFD	4.400	0.183
HFD + Positive	1.350	0.129
HFD + 100	2.425	0.096
HFD + 300	1.950	0.420
HFD + 500	1.400	0.082

Group	Relative expression (fold of normal)	SD
Normal	1.100	0.141
HFD	0.400	0.082
HFD + Positive	0.850	0.058
HFD + 100	0.375	0.096
HFD + 300	0.500	0.082
HFD + 500	0.775	0.050

Values are presented as mean ± SD. Gene expression levels were normalized to the normal group.

Supplementary Table S5. Adipo tissue TNF-α, IL-6, IL-1β, and adiponectin protein expression

Group	TNF-α (pg/mL)	SD
Normal	26.6	2.41
HFD	92.2	13.20
HFD + Positive	65.6	3.97
HFD + 100	92.0	9.14
HFD + 300	76.6	8.38
HFD + 500	57.2	5.45

Group	IL-6 (pg/mL)	SD
Normal	28.4	4.04
HFD	77.0	3.67
HFD + Positive	42.2	2.86
HFD + 100	68.6	4.93
HFD + 300	52.4	1.95
HFD + 500	40.0	5.52

Group	IL-1β (pg/mL)	SD
Normal	23.2	1.30
HFD	33.6	3.21
HFD + Positive	24.2	1.92
HFD + 100	33.8	1.79
HFD + 300	31.6	2.41
HFD + 500	23.4	1.67

Group	Adiponectin (pg/mL)	SD
Normal	26.6	3.51
HFD	12.8	1.92
HFD + Positive	20.8	1.92
HFD + 100	13.8	1.30
HFD + 300	15.2	2.59
HFD + 500	21.8	2.59

Values are presented as mean ± SD. Serum inflammatory cytokines and adipokine levels were measured at the end of the experimental period.

Supplementary Table S6. Serum triglyceride, Total cholesterol, ALT, AST levels

Group	Triglyceride (mg/dL)	SD
Normal	90.4	22.85
HFD	234.6	39.15
HFD + Positive	118.2	11.97
HFD + 100	228.0	38.07
HFD + 300	194.2	9.83
HFD + 500	129.4	26.02

Group	Total cholesterol (mg/dL)	SD
Normal	110.2	17.43
HFD	293.8	19.50
HFD + Positive	130.6	20.54
HFD + 100	288.2	32.78
HFD + 300	286.0	15.35
HFD + 500	163.2	17.75

Group	ALT (U/L)	SD
Normal	39.4	6.91
HFD	126.6	14.62
HFD + Positive	42.0	14.70
HFD + 100	118.8	26.55
HFD + 300	97.6	11.97
HFD + 500	33.2	9.68

Group	AST (U/L)	SD
Normal	88.4	15.19
HFD	229.0	50.15
HFD + Positive	140.0	31.91
HFD + 100	199.6	51.80
HFD + 300	201.0	35.72
HFD + 500	149.2	20.77

Values are presented as mean ± SD. Serum biochemical parameters were analyzed at the end of the experimental period.

Supplementary Table S7. Quantification of lipid droplet area in adipose tissue

Group	Lipid droplet area (%)	SD
Normal	2.5	1.2
HFD	43.3	3.5
HFD + Positive	12.3	2.4
HFD + 100	22.5	1.8
HFD + 300	17.5	2.7
HFD + 500	8.5	1.3

Values are presented as mean ± SD. Lipid droplet area was quantified from histological images and expressed as a percentage of total tissue area.
